# The Ubiquitin Proteasome System in Genome Stability and Cancer

**DOI:** 10.3390/cancers13092235

**Published:** 2021-05-06

**Authors:** Jonathan J. Morgan, Lisa J. Crawford

**Affiliations:** Patrick G Johnston Centre for Cancer Research, Queen’s University Belfast, Belfast BT9 7BL, UK; jmorgan30@qub.ac.uk

**Keywords:** ubiquitination, genome stability, DNA replication, DNA damage, cancer

## Abstract

**Simple Summary:**

Genomic instability is a major driving force of tumour development and evolution. Cells have developed sophisticated regulatory systems to preserve the stability of the genome and defects in these mechanisms can lead to the acquisition of mutations. In this review we look at the role of ubiquitination, a common post-translational modification, in the regulation of genomic integrity.

**Abstract:**

Faithful DNA replication during cellular division is essential to maintain genome stability and cells have developed a sophisticated network of regulatory systems to ensure its integrity. Disruption of these control mechanisms can lead to loss of genomic stability, a key hallmark of cancer. Ubiquitination is one of the most abundant regulatory post-translational modifications and plays a pivotal role in controlling replication progression, repair of DNA and genome stability. Dysregulation of the ubiquitin proteasome system (UPS) can contribute to the initiation and progression of neoplastic transformation. In this review we provide an overview of the UPS and summarize its involvement in replication and replicative stress, along with DNA damage repair. Finally, we discuss how the UPS presents as an emerging source for novel therapeutic interventions aimed at targeting genomic instability, which could be utilized in the treatment and management of cancer.

## 1. Introduction

Genome instability first emerged as a hallmark of cancer in the revised famous article “Hallmarks of cancer: The Next Generation” [1]. From there, research and drug discovery surged to understand this mechanism that underscores both development and progression of cancer. Faithful DNA replication is paramount for maintaining genome integrity and has evolved over millennia, developing sophisticated regulatory systems including DNA damage repair machinery and checkpoint kinases, to ensure that genomic material is passed on to the next generation with the highest levels of fidelity. Often it is alterations in these regulatory systems that pose the biggest threat to genome stability and give rise to the development of many cancers [2,3].

A commonly dysregulated system observed in neoplasms is the ubiquitin proteasome system (UPS). The UPS regulates a myriad of cellular processes that are altered during tumorigenesis, including cell differentiation, cell cycle, cellular homeostasis, DNA replication and DNA repair. The UPS is comprised of three specialized enzymes referred to as: E1, E2 and E3, along with the 26S proteasome, a multi-catalytic ATP-dependent protease complex [4]. The E3 ligases afford specificity to the UPS and aberrant expression or mutation of a number of these enzymes has been linked to malignant transformation [5,6,7,8]. This review focuses on the influence of the UPS, and E3 ligases in particular, on genome stability and how understanding their role in genome integrity could potentially provide novel therapeutic strategies.

## 2. The Ubiquitin Proteasome System

**Figure 1:**
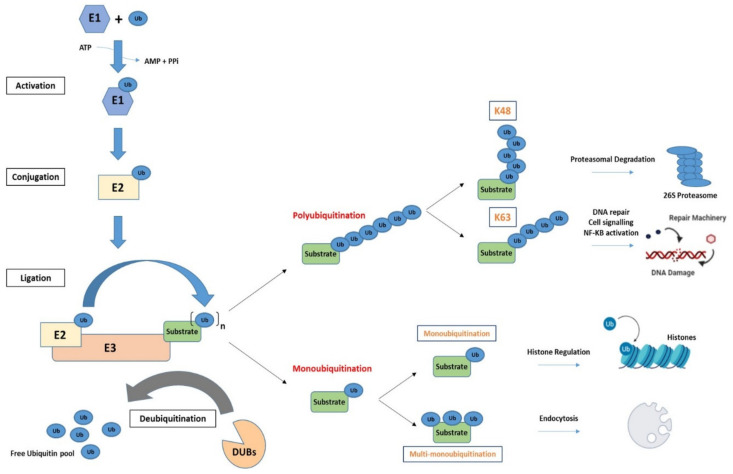
The ubiquitin system. This multicomponent system is made up of E1, E2 and E3 enzymes which facilitate the addition of ubiquitin moieties onto different substrate proteins. Ubiquitin modification are highly specific and are unique for each of the cellular processes they regulate. The deubiquitinating (DUB) family of enzymes allow for the removal of ubiquitin modification and replenishes the free ubiquitin pool within the cell.

As a multi-component regulatory system, the UPS exists in all eukaryotic cells and has been widely studied in the fields of immunology and cancer. It is composed of three types of ubiquitin enzymes and the 26S proteasome [9]. Ubiquitin, a 76 amino acid protein, is highly conserved among eukaryotic organisms and gains the name from its ubiquitous expression in cells. Ubiquitination is one of the most common post-translational modifications (PTM) with ramifications in many cellular processes. It acts as a label or signal to determine the fate and/or function of the substrate protein it marks [10]. The process involves the covalent attachment of an ubiquitin molecule or chain to a lysine (K) residue on the C-terminal of the substrate protein by a cascade of enzymes. The 26S proteasome is a large multi-catalytic protease complex that recognizes and degrades ubiquitinated substrates. It is composed of two distinct complexes—a 20S core particle, capped at one or both ends by a 19S regulatory particle. The 19S regulatory particle functions to recognize ubiquitinated proteins, remove and recycle ubiquitin, unfold the substrate protein and translocate them into the 20S proteasome for degradation. The 20S core particle is a barrel-shaped structure made up of four heptameric rings; the two outer rings are composed of α subunits, which serve as a docking domain for the 19S regulatory particle and the two inner rings are composed of β subunits, three of which contain catalytic sites. Caspase-like, trypsin-like and chymotrypsin-like activities are associated with the β1, β2 and β5 subunits respectively, and confer the ability to cleave after acidic, basic and hydrophobic amino acid residues. The UPS is a highly complex regulatory system that is responsible for the degradation of over 80% of intracellular proteins and; therefore, oversees a myriad of essential processes in the cell. The mechanism and function of the UPS is illustrated below, in Figure 1.

### 2.1. The Process of Ubiquitination

Ubiquitination is performed through the action of three classes of ubiquitin enzymes: an ubiquitin activating enzyme (E1), an ubiquitin conjugating enzyme (E2) and an ubiquitin ligase (E3). The E1 enzyme functions to activate ubiquitin in an adenosine-triphosphate (ATP) dependent manner, forming a high energy thioester bond between a cysteine residue in its active site and the C-terminal of ubiquitin. Ubiquitin is then transferred to a cysteine residue of an E2 enzyme, and in the final step ubiquitin is moved to a lysine residue of a substrate protein by an E3 ligase. The E3 ligase interacts with an ubiquitin-bound E2 enzyme to facilitate the formation of an isopeptide or peptide bond between ubiquitin and a lysine residue of the substrate protein [11,12]. This is a diverse modification where proteins can have one or multiple ubiquitin molecules added to specific lysine residues, whereby both the number and location of the ubiquitin moieties have significance in regard to the form of regulation that the substrate protein will be subject to. There are two known E1 enzymes (UBA1 and UBA6), >30 E2 enzymes and over 600 E3 ligases encoded in the human genome. The addition of ubiquitin moieties to specific residues on a substrate protein is, in part, due to pairings of E2 and E3 enzymes. However, it is the E3 ligase enzymes that predominantly confer specificity to the UPS recruitment of substrate proteins [12].

E3 ligases are classified into three main groups based on their structure and function: Really Interesting New Gene (RING), Homologous to E6-AP Carboxyl Terminus (HECT) and RING-between-RING (RBR). RING finger E3 ligases constitute the largest class and are characterised based on the presence of a RING domain, a type of zinc finger, that confers E3 ligase activity by binding to a ubiquitin-loaded E2 and mediating the direct transfer of ubiquitin to a substrate protein. RING E3 ligases function either as monomers, homo/heterodimers or large multi-subunit complexes, such as the Cullin-RING ligases (CRLs), which generally comprise a RING E3 ligase, a Cullin scaffold and substrate recognition protein [13]. HECT E3 ligases contain an N-terminal substrate-binding domain and a C-terminal HECT domain containing a catalytic cysteine that accepts an ubiquitin molecule from an E2 before conjugating ubiquitin to a substrate protein [14]. RBR E3 ligases contain two RING domains (RING1 and RING2) with an InBetweenRING (IBR) domain between them, and share common features with both RING and HECT E3s. The RING1 domain binds to ubiquitin-bound E2 and transfers ubiquitin onto a catalytic cysteine on the RING2 domain before its conjugation to a substrate protein [15].

### 2.2. Ubiquitination Is a Diverse Modification

When it was first described, ubiquitination was thought to be solely a post-translational modification that labelled proteins for degradation via the 26S proteasome by the addition of K48-linked ubiquitin chains. However, numerous additional linkages have been identified that play central roles in diverse biological processes. Currently there are seven known types of lysine associated ubiquitin linkages (K6, K11, K27, K29, K33, K48 and K63) and one methionine residue (M1) found at the N-terminal. In the most elementary form of ubiquitination, one ubiquitin molecule is added to a lysine residue of a substrate protein. This is referred to as mono-ubiquitination and has been predominantly linked with the regulation of histones [16,17]. Further, multi-mono-ubiquitination where single ubiquitin molecules are added to multiple lysine residues has been linked with endocytosis [18]. Additionally, further complexity and versatility in the system has been identified through the discovery of both homotypic and heterotypic chains. Homotypic, refers to chains in which ubiquitin molecules are connected through the same lysine residue while, heterotypic chains are conjugated through different lysine residues. [19].

The type of ubiquitin linkage determines the form of regulation placed on the substrate protein. K6 ubiquitin linkages remain poorly characterised; however, they have been implicated in the DNA damage response, with K6 ubiquitin linkages found on the tumour suppressor E3 ligase breast cancer 1 (BRCA1) and its substrate proteins [20]. K11 ubiquitin linkages have been associated with both signals for proteasome degradation and regulation of cell cycle progression. For example, the multi-subunit RING E3 ligase, anaphase-promoting complex/cyclosome (APC/C) utilizes K11 ubiquitin conjugation during mitosis, as the cell transitions from metaphase to anaphase [21]. K27 ubiquitination is reported to be an important linkage to promote DNA damage response (DDR) mediators. Gatti et al. found that activation of the DDR at double-strand breaks (DSBs) requires K27 ubiquitination of the histone 2A (H2A) by the E3 ligase RNF168 [22]. K29 and K33 ubiquitin modifications have been associated with many roles within the cell including autophagy, protein trafficking, stress responses and cell cycle regulation [23,24]. The methionine-linked ubiquitin modification (M1) or linear ubiquitin chains are added to the N-terminal of a substrate protein by the linear ubiquitin chain assembly complex (LUBAC), the only known E3 ligase capable of the addition of these linear chains, and are well characterised for their role in the activation of the transcription factor nuclear factor kappa B (NF-κB) [25]. The best characterised ubiquitin modifications are the addition of K48 and K63 poly-ubiquitin linked chains. While the K48-linked ubiquitin chains result in proteolytic degradation by the 26S proteasome, K63-linked modifications are responsible for mediating protein-protein interactions and have been associated with the DDR [26]. For example, the E3 ligase TRAF6 has been shown to assist in the trafficking of DNA repair proteins to sites of DNA damage through K63-linked poly-ubiquitination [27].

In common with most post-translational modifications, ubiquitination is reversible, and ubiquitin removal is carried out by the actions of a complex family of cysteine protease deubiquitinating enzymes, referred to as DUBs. These enzymes act to remove ubiquitin or remodel ubiquitin chains on substrate proteins allowing for the generation of free ubiquitin molecules that can be then recycled by the UPS in other cellular processes. The balance between ubiquitination and deubiquitination acts to maintain protein homeostasis and protein activities [28].

## 3. DNA Replication and Replicative Stress: UPS Surveillance of the Genome

In simple terms, replication is the duplication of the genome that starts with DNA double helix and proceeds in a semi-conservative fashion, whereby each strand of the double helix acts as a template for the creation of two new strands; the finished product is two double helices, containing one old strand and one new strand. In reality replication is a complex process that is orchestrated by proteins that act almost simultaneously, and is controlled by a group of enzymes, checkpoint kinases, that regulate cycle-dependent kinase activity and respond to perturbations on DNA that risks the integrity of the genome [29].

Replication begins with the assembly of pre-replication complexes (pre-RCs) at multiple sites across the genome. Double stranded DNA is unwound at these sites by DNA helicases to form a replication fork containing two single-stranded DNA templates which are subsequently utilized by DNA polymerases to replicate the DNA [30]. Termination of replication occurs upon the convergence of replication forks; DNA synthesis is completed and so the replisome dissociates [31]. It is important for both initiation and termination of replication to be tightly regulated to ensure for timely cell cycle progression and faithful duplication of the genome.

### 3.1. Initiation of Replication

**Figure 2:**
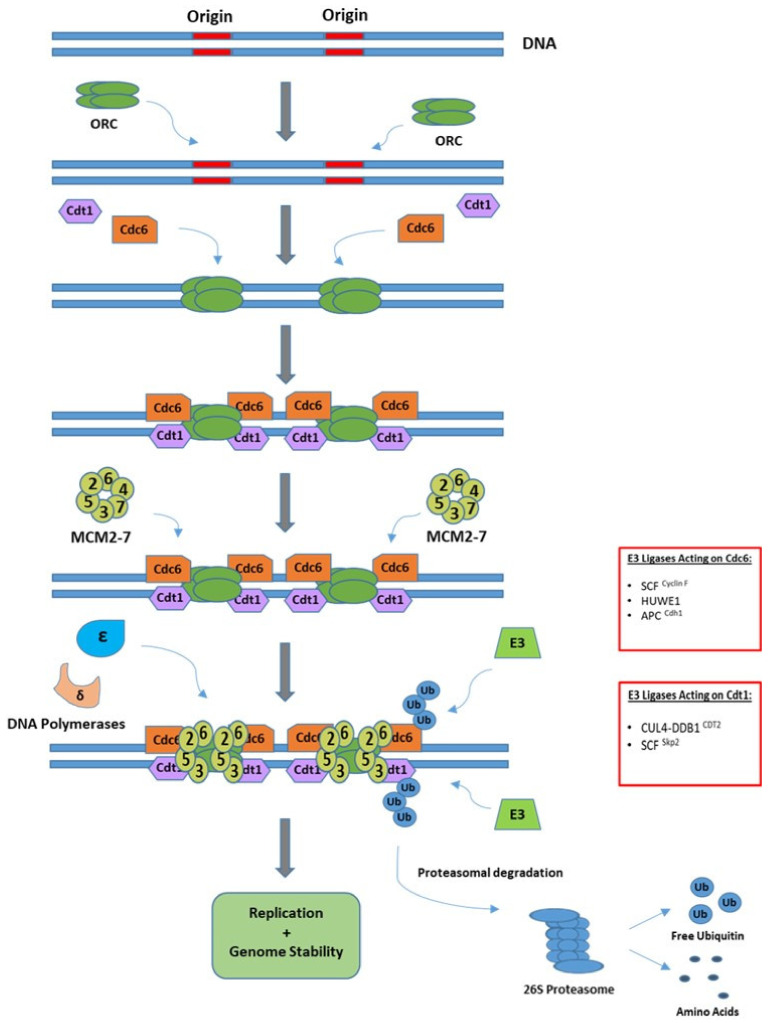
Initiation of DNA replication. Licensing of origins requires the loading of origin replication complex (ORC) proteins; Cdt1 and Cdc6 for the recruitment of helicases and other components of the replisome. The ORC proteins are subsequently removed upon entry to S-phase by proteolytic degradation mediated by the ubiquitin proteasome system thereby preventing re-replication.

Initiation of replication begins at specific genomic sites, known as replication origins and can be divided into two phases, referred to as licensing and firing. Licensing occurs when the cell cycle is progressing from M to G_1_ phase and involves the assembly of pre-RCs at replication origins. Pre-RCs are formed when the origin recognition complex (ORC), made up of six subunits (ORC1–6), recognizes and binds to replication origins. This promotes recruitment of CDT1 and CDC6, which in turn allows for the helicase mini-chromosome maintenance complex (MCM2–7) to be loaded onto DNA to form a pre-RC [30]. Origin activation, or firing, subsequently occurs upon entry to S phase, whereby MCM2–7 is activated by the kinases CDK and DDK, triggering the recruitment of CDC45 and the GINS complex to form the functional helicase CMG (CDC45/MCM2–7/GINS) [32]. Activation or firing of origins is reliant on timely coordination of each of the components to allow unwinding of the double-strand helix and to prevent re-replication of DNA. Separation of origin licensing and origin firing into different phases of the cell cycle is the key replication-limiting mechanism and it is regulated in part through cell cycle-dependent ubiquitination of key replication factors, including CDC6, CDT1 and ORC1 [33]. Ubiquitin-mediated degradation of CDC6 by the E3 ligase complex APC/C^Cdh1^ in early G_1_ prevents its accumulation until late G_1_ where it is required to form pre-RCs [34]. The Cullin RING Ligase CRL4 is recruited to replication origins in G_1_ by the CRL4 substrate receptor replication initiation determinant protein (RepID) to facilitate initiation of replication [35]. CRL4 containing CDT2 as a substrate recognition subunit targets CDC6 for degradation once cells enter S-phase, and the SCF (SKP1-Cullin1-F-Box protein) ubiquitin ligase complex with the substrate receptor Cyclin F (SCF^Cyclin F^) promotes the degradation of CDC6 in late G_2_ and early M-phase, thus preventing origin relicensing [36,37]. This pre-RC protein is also ubiquitinated and marked for degradation upon DNA damage by the large HECT-E3 ligase HUWE1 in S and G_2_ phases, when the APC/C^Cdh1^ ligase complex is inhibited. Control of CDC6 protein levels during later cell cycle stages by HUWE1 is pivotal in maintaining genome integrity by preventing replication of DNA lesions [38,39]. The removal of CDT1 from DNA replication origins is mediated by the SCF^Skp2^ E3 ligase complex at the G_1_ to S phase transition and, subsequently, by CUL4^CDT2^ in S-phase to ensure it is not available for relicensing origins [40]. CUL4^CDT2^-mediated degradation of CDT1 is dependent on its binding to proliferating cell nuclear antigen (PCNA), which directly interacts with CDT1 to promote its ubiquitination [41]. Conversely, CDT1 is stabilized in G_1_ by the APC/C ^Cdh1^-mediated degradation of Geminin, an inhibitor of CDT1 [42]. Finally, after origin firing, ORC1, the largest subunit of the ORC complex, is ubiquitinated and degraded by SCF^Skp2^ [43]. A schematic of the initiation of replication, along with regulatory E3 ligases is given in Figure 2.

### 3.2. Elongation and Termination of Replication

Following origin firing, a number of additional replication proteins, including replication protein A (RPA), PCNA and DNA polymerases, are recruited to nascent replication forks to begin DNA synthesis. This involves the creation of new DNA strands that are incorporated into a double helix with the original template strand, the two strands are joined by hydrogen bonds and are subject to Chargaff’s law where; adenine (A) only binds with thymine (T) and cytosine (C) will always bind to a guanine (G). The creation of these hydrogens bonds is performed largely by the DNA polymerases ε and δ in a 5′ to 3′ bidirectional manner, whereby the two new strands are synthesized simultaneously. The leading strand is synthesized continuously in a 5′–3′ direction towards the replication fork, while the lagging strand is synthesized discontinuously by small DNA fragments referred to as Okazaki fragments [44]. The synthesis of the leading and lagging strands continues until two replication forks converge [45]. Termination of replication requires the disassembly of the replication machinery from chromatin, and is regulated by ubiquitination. Polyubiquitination of MCM7, by the E3 ligase complex CRL2^LRR1^, recruits the ATPase VCP/p97 to remove MCM7 from chromatin leading to disassembly of the MCM complex [46,47,48,49].

### 3.3. Ubiquitination at Stalled Replication Forks

Replication can also be terminated or stalled prematurely when replication forks encounter obstacles such as DNA damage, DNA-protein crosslinks, DNA-RNA hybrids and replication stress. Arresting replication and the formation of a stalled fork serves to prevent unfaithful DNA replication, but if it persists the stalled fork can result in the formation of double-strand breaks and further compromise the integrity of the genome. Cells have developed sophisticated mechanisms to overcome replication fork barriers including fork reversal, translesion synthesis (TLS) and template switching (TS). Ubiquitination plays a crucial role in the regulation of fork stability and DNA damage response at stalled forks, the key players in this are discussed below.

#### 3.3.1. Regulation of RPA

At stalled replication forks, the replicative DNA helicase and DNA polymerases are uncoupled from the DNA generating regions of ssDNA. This ssDNA is rapidly bound by RPA which serves as a signalling platform to recruit factors involved in replication stress and DNA damage responses, as well as the subsequent restart of stalled forks. RPA is a heterotrimeric protein composed of three subunits: RPA70, RPA32 and RPA14, and can be ubiquitinated at multiple lysines upon replication fork stalling [50].

Optimal loading of RPA onto ssDNA at stalled forks and subsequent modification of RPA requires the timely degradation of the replication stress response regulator SDE2. During replicative stress, SDE2 is first cleaved by PCNA to generate a C-terminal fragment known as SDE2^Ct^. SDE2^Ct^ is recognized and polyubiquitinated by the UBR1/2 E3 ligase and subsequently extracted and degraded via the VCP/p97 segregase complex. Cells lacking SDE2^Ct^ fail to induce a ssDNA-RPA platform, leading to defects in PCNA-dependent DNA damage bypass and stalled fork recovery [51].

To date, there are several E3 ligases that are known to modulate RPA’s ubiquitination during replication stress, including RFWD3 and PRP19. RFWD3 has recently been shown to facilitate the ubiquitination of RPA subunits both for normal DNA replication and in response to replicative stress. In unperturbed cells, RFWD3 is recruited to and stabilized at replication forks by PCNA, where it targets RPA for proteasomal degradation to allow fork progression to proceed [52]. Cells lacking RFWD3 display an accumulation of RPA and increased frequency of stalled replication forks. A number of roles have been reported for RFWD3-mediated ubiquitination at stalled replication forks. RFWD3 has been shown to promote non-proteolytic ubiquitination of all three RPA subunits to promote homologous recombination (HR)-dependent fork repair and restart [53]. Inano and colleagues subsequently found that RFWD3 facilitates HR through polyubiquitination of both RPA and RAD51, leading to VCP/p97-mediated degradation [54]. Furthermore, RPA-mediated recruitment of RWD3 to stalled replication forks is essential for the repair of DNA interstrand crosslinks (ICLs), lesions that inhibit DNA strand separation and therefore block replication. Mutations in RFWD3 lead to defects in ICL repair by disrupting RPA-RFWD3 binding at ICL—induced stalled replication forks and have been associated with Fanconi anaemia (FA), a rare genetic disorder characterised by genomic instability and predisposition to cancer [55]. Mutations in BRCA2, which functions in replication fork stability and HR, are also associated with FA and RWFD3 has been shown to affect stalled fork stability in BRCA2 mutant cells. In the absence of BRCA2, RPA is hyperubiquitinated by RFWD3 at stalled forks, contributing to fork instability and collapse [56].

PRP19 is an essential U-BOX E3 ligase that is well known for its role in pre-mRNA processing. While RFWD3 is constitutively associated with RPA, PRP19 binds and ubiquitinates RPA after DNA damage [57]. In the absence of this enzyme, cells exhibit a heightened sensitivity to inducers of replication-stress including UV and hydroxyurea (HU). Furthermore, knockdown of PRP19 leads to reduced K63-linked ubiquitination of RPA 70 and 32 subunits during induced replication stress with an associated attenuation of ataxia telangiectasia and Rad3-related (ATR) signalling, alongside a subsequent decrease in the abundance of phosphorylated ATR substrates RPA and checkpoint kinase 1 (Chk1) [58].

#### 3.3.2. PCNA Ubiquitination

Modification of PCNA by ubiquitin plays a crucial role in rescuing stalled replication forks. Monoubiquitination of PCNA at K164 by the E2-E3 ubiquitin ligase complex Rad6-Rad18 promotes error-prone TLS mediated replication, a process which uses TLS polymerases to promote replication across the DNA lesion [59]. Meanwhile, K63-linked polyubiquitination of PCNA at K164 by helicase-like transcription factor (HLTF) promotes TS, which employs the use of the newly synthesized daughter strand as a template to bypass the DNA lesion [60]. K63-linked polyubiquitination of PCNA is also important for replication fork reversal and restart. The translocase ZRANB3 is recruited to K63-linked PCNA to stabilize stalled forks and facilitate replication restart [61]. The HECT E3 ligase HUWE1 has also been reported to relieve replicative stress in cells by facilitating fork restart through its interaction with PCNA. Choe et al. [62] demonstrated that HUWE1 binding to PCNA at stalled forks resulted in the recruitment of DNA repair machinery through HUWE1-mediated mono-ubiquitination and subsequent phosphorylation of the DNA damage marker H2AX. The DNA repair proteins including BRCA1 and BRCA2 allow repair of DNA and restart of the replication fork; HUWE1 promotes the repair and ultimately the restart of stalled forks aiding the integrity of the genome that would otherwise be compromised with creation of DNA breaks from prolonged fork stalling [63]. PCNA is also ubiquitinated by several other E3 ligases, including CDT1 and BRCA1, and likely serves as an additional control mechanism to reduce the number of stalled forks and limit the incidences of DSBs [64].

#### 3.3.3. TRAIP-Mediated Regulation of Replisome Stability

TRAF-interacting protein (TRAIP) is a replisome-associated RING E3 ligase with important roles in replication and in promoting genomic stability. In response to replication blocking lesions such as ICLs or DNA-protein crosslinks (DPCs), TRAIP-mediated ubiquitination promotes the completion of DNA replication in a number of ways. ICLs can be repaired by two pathways: The FA pathway can create a DSB that is repaired through HR, or the DNA glycosylase NEIL3 can unhook or cleave the cross link. TRAIP functions upstream of these pathways and can determine the pathway choice. Convergence of replication forks at a crosslink triggers TRAIP-mediated ubiquitination of the CMG helicase. Short ubiquitin chains recruit NEIL3 to unhook the ICL thereby allowing completion of replication. Alternatively, the ubiquitin chain can be extended to facilitate CMG unloading by VCP/p97, enabling the FA pathway and subsequent HR repair of the lesion [65]. DPCs block progression of DNA replication and arrival of a replication fork at a DPC triggers TRAIP ubiquitination of the DPC, in turn promoting CMG bypass of the lesion and proteasomal degradation of the DPC [66]. Another important function of TRAIP in preserving genome stability is by triggering replisome unloading in mitosis. Failure to rescue stalled forks or repair DNA damage can result in unreplicated DNA persisting into mitosis, which can lead to mitotic defects including chromosomal rearrangements. TRAIP promotes replisome disassembly in mitosis through K6- and K63-linked ubiquitination of MCM7, leading to CMG unloading by VCP/p97 [67].

#### 3.3.4. R-Loop-Induced Stress

**Figure 3:**
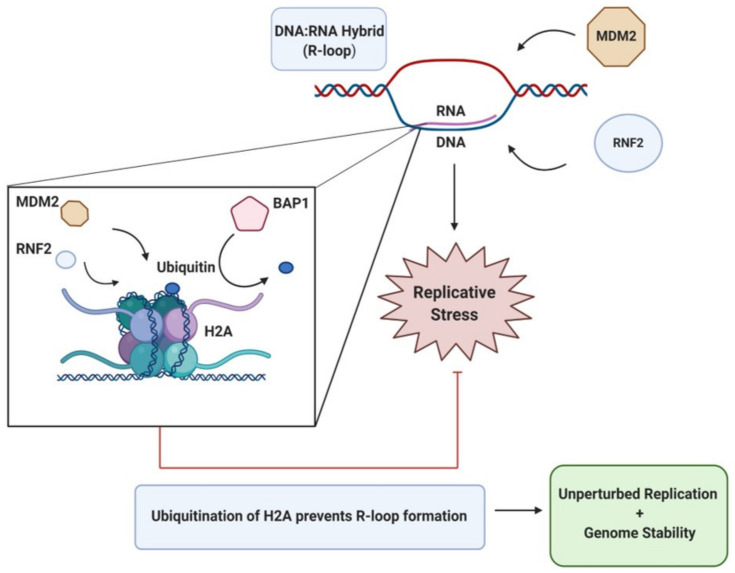
Removal of R-loops. The formation of DNA:RNA hybrids are a common occurrence and pose a threat replication and to genome integrity. The removal of these R-loops is facilitated by the mono-ubiquitination of histone H2A (K119) by the E3 ligases MDM2 and RNF2 and by its timely deubiquitination by BAP1. It is this balanced ubiquitination of H2A that suppresses R-loop formation and minimizes genomic instability.

The formation of DNA:RNA hybrids, referred to as R-loops, pose a threat to fork progression and ultimately act as a source of replication stress that can contribute to genomic instability. While the exact mechanisms of how R-loops are formed and add to instability in the genome is not yet delineated, there is evidence of UPS-mediated regulation of R-loops during replication to counteract replicative stress (Figure 3). By their nature R-loops leave single-strand DNA (ssDNA) exposed and susceptible to harmful lesions with an additional risk of transcription-associated mutagenesis [68]. Two E3 ligases, MDM2 and RNF2, have been reported to act to prevent the formation of R-loop structures that would impair replication. They achieve this by promoting the mono-ubiquitination of H2A at K119, with coordinated deubiquitination by the DUB enzyme, BAP1, which removes the ubiquitin modification when appropriate [69,70]. It is this balance of ubiquitination and deubiquitination that supports DNA replication and prevents the formation of R-loops. Pharmacological targeting of MDM2 is currently under investigation with the aim of potentially sensitising cancer cells to topoisomerase inhibitors, a drug that induces R-loop formation [62,63]. The rationale for this might be explained by the findings of Klusmann et al. [70], where depletion of MDM2 left cells predisposed to the occurrence of these DNA:RNA structures and thereby the genomic instability that they promote. Interestingly, overexpression of MDM2 has a similar effect with cells exhibiting heightened levels of replication stress and cell cycle arrest.

## 4. The UPS Facilitates the DNA Damage Response

A major obstacle for genome stability is avoiding the acquisition of damage during S phase [71]. The cell has developed sensory bodies known as checkpoint kinases that initiate cascades of cellular signalling that halt replication and cell cycle progression, allowing the recruitment of DNA repair machinery to the site of damage with goal of repairing damage and restoring normal cell cycle. In response to genotoxic stress, DNA damage is recognized by the kinases ATM and ATR which coordinate a network of cellular processes to maintain genomic integrity, including the DDR, made of multiple pathways for the detection and repair of different types of DNA damage [72]. Specific repair mechanisms are designated for single-strand breaks (SSBs) including base excision repair (BER) and nucleotide excision repair (NER) pathways, while in the case of DSBs the homologous recombination (HR) and non-homologous end joining (NHEJ) pathways are activated [73]. These repair pathways are present at different points in the cell cycle ensuring minimal amounts of damage are replicated and passed on. Similar to any physiological process, post-translation modifications such as ubiquitination contribute to the tight control of DDR signalling and prevent aberrant activation of DNA damage repair. Ubiquitin-mediated regulation of individual DDR pathways has been extensively reviewed and here we focus predominantly on ubiquitin regulation of upstream DNA damage sensing and DDR signalling via ATM and ATR.

### 4.1. UPS Regulation of ATR-Mediated Repair

A broad spectrum of DNA damage stimuli, such as UV radiation, replication stress and interstrand DNA crosslinking agents, result in activation of ATR, a kinase that activates and recruits a number of substrates including the protein kinase Chk1 (checkpoint kinase 1). ATR-Chk1 signalling promotes the degradation of CDC25A phosphatase via the UPS and prevents the de-phosphorylation of cyclin dependent kinases (Cdc2/cyclinB1) leading to a halt in replication and cell cycle progression. Two E3 ligase complexes, APC/C^Cdh1^ and SCF^βTrCP1/2^, play a role in regulation of CDC25A protein levels in cells upon DNA damage in a cell cycle dependent manner [74]. During late mitosis and G_1_ phase the APC/C^Cdh1^ ligase is responsible for regulating the abundance of CDC25A, and the E3 ligase labels it with ubiquitin by recognition of a specific sequence known as a KEN-box motif found on the N-terminus of the protein. While during S and G_2_ phase, CDC25A is modulated by an SCF ligase containing a βTrCP1 or βTrCP2 F-box protein [74]. The SCF^βTrCP1/2^ ligase recognizes phosphorylated CDC25A, ubiquitinates it thus promoting its degradation via the 26S proteasome. CDC25A is predominantly phosphorylated at Ser76 by the ATR-Chk1 axis, in collaboration with a DNA binding protein known as claspin and the Rad9-Rad1-Hus1 complex. Other paralogs of CDC25, including CDC25B and CDC25C, are regulated by Tribbles homolog 2 (TRIB2), a member of Tribbles family of serine/threonine psuedokinases, which promotes their ubiquitination and degradation. TRIB2 is thought to act as an adaptor protein working in conjunction with a currently unknown E3 ligase to promote the addition of K48 ubiquitin linkages and thereby regulate the G_2_/M DNA damage checkpoint [75,76]. Mutations in both βTrCP1 and βTrCP2 have been reported in cancers potentially leading to stabilisation and accumulation of CDC25A and subsequent replication stress and genomic instability [77].

### 4.2. UPS Regulation of ATM-Mediated Repair

While ATR can be activated by a range of stimuli, ATM is predominantly activated by DSBs [78]. ATM is brought to the site of the DSB by the MRN complex (MRE11-RAD50-NBS1) in an ubiquitin-dependent manner. The E3 ligase Skp2 attaches K63-linked polyubiquitin chains to the MRN subunit NBS1, which in turn recruits ATM for activation [79,80]. Upon recruitment of ATM to the site of the DSB, it phosphorylates a plethora of substrates, including the protein kinase Chk2, histone H2AX and tumour suppressor p53, to mediate effects on DNA repair, cell cycle arrest and apoptosis. Phosphorylation of Chk2 serves to amplify and expand ATM-mediated signalling.

The tumour suppressor p53 is stabilized upon DNA damage and plays a central role in maintaining genome stability. ATM phosphorylates both p53 and its inhibitor, the E3 ligase MDM2 and this serves to both activate p53 and protect it from proteasomal degradation via MDM2 polyubiquitination [81]. Once activated p53 can facilitate DNA repair by inducing a cell cycle arrest allowing time for DNA repair, and it can also directly impact many of the DDR signalling pathways [82]. P53 is frequently mutated or deleted in cancer and wild-type p53 expression can also be downregulated through MDM2 overexpression, leading to genomic instability.

Phosphorylation of H2AX on Ser139 initiates the recruitment of DNA repair complexes, in part through ubiquitin signalling. Phosphorylated H2AX, or γH2AX, recruits the mediator of DNA damage checkpoint protein 1 (MDC1) to sites of DNA damage [83]. Here MDC1 undergoes ATM-mediated phosphorylation and in turn recruits the E3 ligases RNF8 and RNF168 [84]. RNF8 facilitates the K63 linked poly-ubiquitination of H1 linker histones at the site of double-strand breaks and that in turn mediates the mono-ubiquitination of H2A-type histones on K13 and K15 by RNF168 [85]. The ubiquitination of these histones results in an eventual accumulation of repair proteins p53 binding protein 1 (53BP1) and BRCA1. These proteins have reciprocal roles in the repair of DSBs, 53BP1 is associated with NHEJ repair of the DSB, while BRCA1 promotes HR-mediated repair. The actions of RNF8 and RNF168 are instrumental in the cells ability to repair DSBs with studies in mice showing that knockout of either of these ligases predisposes cancer development [86,87]. The E3 ligase RNF4 is another key player in DSB repair through its effects on MDC1 and downstream DDR factors. RNF4 is a Small Ubiquitin-like Modifier (SUMO)-targeted ubiquitin ligase (STUbL) that specifically recognizes and ubiquitinates proteins modified with SUMO. While MDC1 is required for the recruitment of DDR factors, its removal from DSBs is required for HR-mediated repair. SUMOylation of MDC1 by the SUMO E3 ligases PIAS1 and PIAS4, recruits RNF4 to promote turnover of MDC1 via the proteasome, thereby facilitating access of other DDR factors to sites of damage [88].

**Figure 4:**
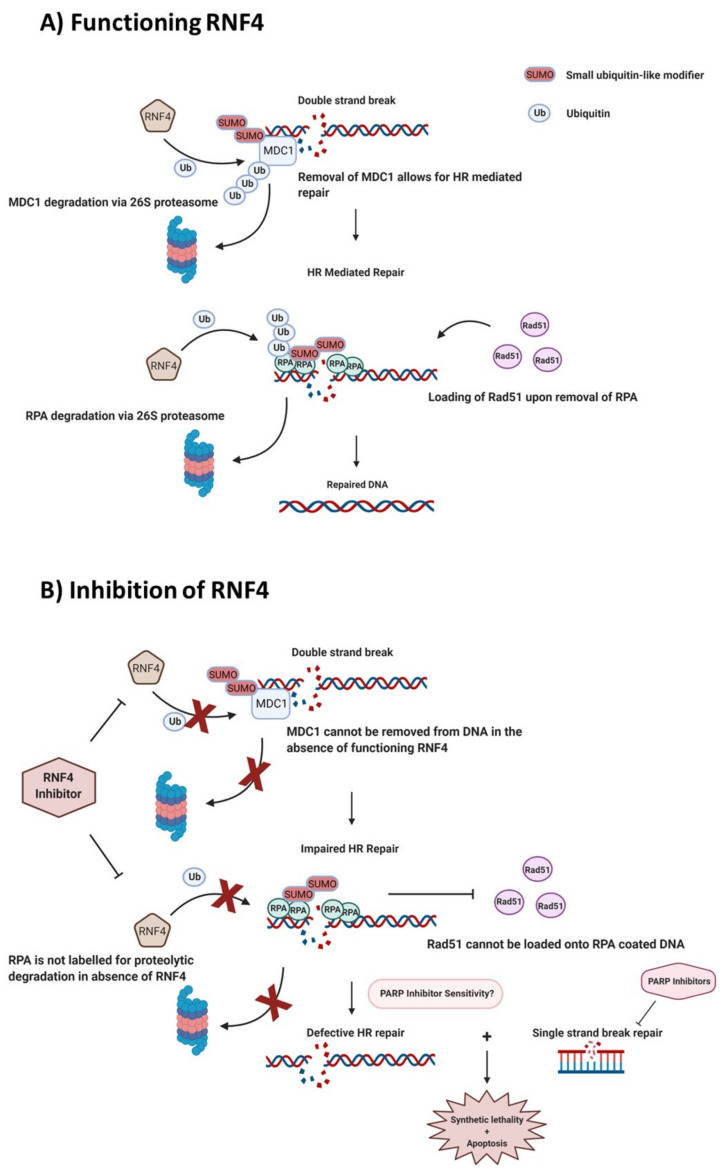
Inhibition of RNF4. (**A**) Functioning RNF4. The E3 ligase RNF4 recognizes small ubiquitin-like modifier (SUMO)-ylated mediator of DNA damage checkpoint protein 1 (MDC1) and replication protein A (RPA), labelling them for proteasomal degradation, resulting in; activation of Ataxia telangiectasia mutated kinase (ATM) and loading of Rad51 onto DNA. RNF4 mediated ubiquitination allows for successful HR repair. (**B**) Inhibition of RNF4 results in a homologous recombination (HR) defect, leading to an increased sensitivity to PARP inhibitors.

The loading of HR proteins, including RPA and RAD51, on to the DNA is an important part of this DDR pathway. During HR-mediated repair of DSBs, ssDNA is coated with RPA subunits to prevent ssDNA from binding to itself before RAD51 can be recruited. In order for RAD51 to be loaded on to the DNA by its binding protein BRCA2, the RPA must first be removed [89]. RNF4 also plays a key role in regulating RPA turnover and BRCA2-mediated RAD51 loading. During HR, PIAS1 and PIAS4 SUMOylate RPA70, which recruits RNF4 to RPA70, leading to ubiquitin-mediated degradation of RPA70 [90]. In the absence of RNF4, BRCA2 is not efficiently recruited to sites of DNA damage and cells exhibit a HR repair defect, similar to the HR deficiency that results from mutations in the BRCA1 or BRCA2 genes, a so-called ‘BRCAness’ phenotype [90]. While there are adverse effects of a HR defect such as increased mutagenesis and genomic instability there is also a targetable therapeutic vulnerability in tumour cells harbouring a HR repair defect. BRCA1/2 mutants or tumours displaying BRCAness exhibit a heightened sensitivity to PARP inhibition by eliciting cell death via a synthetic lethality mechanism [91,92]. Inhibition of PARP in HR-deficient cells prevents repair of SSBs in an already DSB repair defective setting leading to replication fork collapse, unrepaired DNA damage and cytotoxicity. Inhibition of RNF4 could offer an additional mechanism to sensitize cancer cells to PARP inhibition (Figure 4).

## 5. Therapeutic Interventions

Targeting the UPS has been at forefront of many biomedical research laboratories across the world for the last few decades, with development of the first in class proteasome inhibitor bortezomib and additional second-generation proteasome inhibitors swiftly following. More recently there has been an influx in research into components of the UPS that precede the proteasome including drugs designed to target ubiquitin-conjugating (E2) and ubiquitin ligase enzymes (E3), as well as therapies focused at the DUBs. Here we focus on those aimed at DNA replication and repair pathways, with an overview of compounds in clinical development presented in Table 1.

### 5.1. The Proteasome

Inhibition of proteasome function is established as a powerful anti-cancer strategy for some haematological malignancies. Bortezomib was introduced into the clinic for the treatment of multiple myeloma in 2003 and mantle cell lymphoma in 2006 and has contributed towards improved survival for many patients [93,94]. Following the success of bortezomib, second generation proteasome inhibitors carfilzomib and ixazomib were subsequently approved for clinical use [95,96] and additional proteasome inhibitors (oprozomib, marizomib) are in clinical trials [97,98]. While the inhibitors differ in their pharmacodynamics properties, the key molecular target of all of the inhibitors is the β5 catalytic subunit [99]. Proteasome inhibitors are largely thought to exert their anti-cancer effect by inducing an acute proteotoxic effect. However, this proteotoxicity has been shown to also have an implication for the DDR. When the proteasome is inhibited this results in a reduction of free ubiquitin in the nucleus, abrogation of H2AX ubiquitination and decreased recruitment of BRCA1 and Rad51 to sites of DSBs, leading to impaired HR. This so called ‘BRCAness’ phenotype, induced by proteasome inhibitors, sensitizes cells to PARPi in a similar manner to the synthetic lethality observed with PARPi in BRCA-deficient tumours [100].

### 5.2. MDM2

The tumour suppressor p53 is the most frequently mutated gene in cancer and is fittingly the most studied human gene [101]. Playing a central role in the DDR it has been dubbed the guardian of the genome and has an influence in DNA repair, cell cycle and apoptosis. It is heavily regulated by the UPS, predominantly by ubiquitin-mediated degradation by MDM2, but also through ubiquitination by the E3 ligases HUWE1, p53-induced RING H2 (Pirh2) and constitutive photomorphogenesis protein 1 homolog (COP1), among others [102]. One of the major avenues of research for targeting p53 is using small molecule inhibitors of MDM2. MDM2 is overexpressed in many malignancies including lung, liver, colorectal cancers and many blood neoplasms. The first selective inhibitors of MDM2, were the nutlins or imidazolines derivatives that function as competitors of p53-MDM2 binding [103]. The consequence of this reduced p53-MDM2 binding results in an accumulation of p53 and its substrates and a subsequent increase in apoptosis. The nutlins competitively bind to the hydrophobic pocket of MDM2 by mimicking the three crucial amino acids required for p53 binding (Phe19, Trp23, Leu26) determined by crystallographic studies [104]. Another compound developed to inhibit MDM2 is 5-deazaflavin, which targets the RING finger domain of the protein to obstruct its E3 ligases activity. This results in increased p53 levels due stabilisation of the protein, with an associated increase in p53-mediated apoptotic activity. Deazaflavin analogues function to promote p53 activity by hampering MDM2s ability to ubiquitinate both p53 and itself [105]. Development of inhibitors of MDM2 is a promising strategy not only for its anti-tumour effect but also to overcome chemotherapy resistance, a common clinical problem. Preclinical and early clinical studies have been encouraging and a growing number of MDM2 inhibitors are undergoing clinical evaluation [106].

### 5.3. The Anaphase Promoting Complex

Another potential therapeutic target in the UPS is the anaphase promoting complex (APC/C). The APC/C uses one of two coactivators for substrate recognition, CDC20 and CDH1, which activate the complex at distinct phases of the cell cycle. APC/C^CDC20^ primarily controls progression from metaphase to anaphase and mitotic exit, while APC/C^CDH1^ and is primarily active through mitotic exit and early G_1_ [107]. Two small molecule inhibitors of APC/C have been developed, pro-TAME and Apcin, which function using distinct mechanisms. Apcin disrupts the interaction of CDC20 with APC/C substrates, while pro-TAME blocks the activity of both APC/C^CDC20^ and APC/C^CDH1^. A number of pre-clinical studies have demonstrated an anti-cancer effect for these compounds, with a combination of Apcin and pro-TAME eliciting a greater effect than either compound alone [108].

### 5.4. Cullin-RING Ligases

The CRL protein family are the largest family of multicomponent E3 ligases and are involved in the regulation of many biological processes. Activation of CRLs requires the conjugation of NEDD8 to a key lysine residue at the C-terminal of Cullins, a process similar to ubiquitination, referred to as neddylation. A number of CRLs are involved in the regulation of DNA replication, including the SCF complex and CRL4. One promising inhibitor of CRLs is MLN4924, a small molecule inhibitor of the NEDD8-activating enzyme (NAE) [109]. MLN4924 exhibits anti-cancer effects in numerous cell types and has been demonstrated to stabilize the replication licensing factor CDT1, through inhibition of SCF and CRL4, leading to re-replication, DNA damage and eliciting a G_2_ cell cycle arrest [110]. MLN4924 is currently undergoing early-phase clinical evaluation across a range of cancer types (Table 1).

In addition to general CRL inhibition, another strategy under investigation is specific inhibition of the SCF subunit SKP2. The F-box protein SKP2 is overexpressed in many cancers and is associated with an inferior prognostic outcome in gastric, colon and breast cancers [111,112]. It negatively regulates the abundance of cyclin-dependent kinase inhibitors including p27, p21 and p57 and so it is not surprising that overexpression of this SCF-E3 ligase complex could result in uncontrolled cell cycle progression. The continuous replication of unchecked DNA allows harmful mutations to be passed on without correction and results in loss of genomic stability and ultimately tumorigenesis. A small molecule inhibitor of SKP2, known as compound 25, was identified through an in silico screen [113]. Compound 25 was found to significantly attenuate the Skp1-SKP2 interaction and displayed synergy with other chemotherapeutics both in vitro and in vivo. More recently, Li et al. [114], reported that treatment with SMIP004, an additional SKP2 inhibitor, led to an increased sensitivity to radiation in human breast cancer cell lines in vitro, with similar results obtained with breast cancer cell xenografts.

### 5.5. Hijacking an E3 Ligase

**Figure 5:**
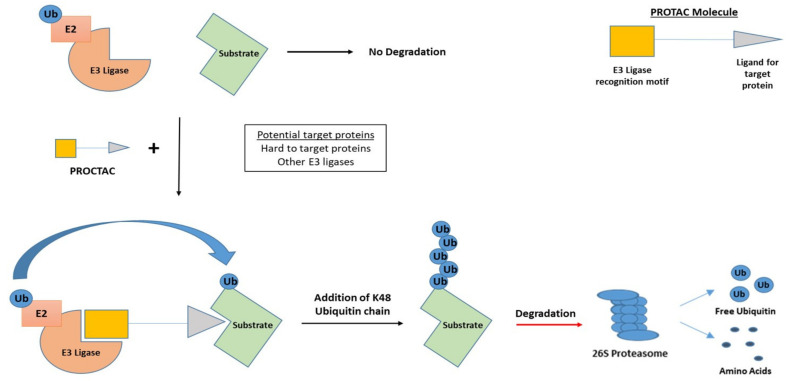
The development of protein-targeting chimeric molecules (PROTACs). Small molecules possessing an E3 ligase recognition motif and a ligand designed for a specific target protein. PROTACs recruit E3 ligases and acts as a bridge between the E3 ligase and target protein, allowing the addition of K48-linked ubiquitin chains and subsequent proteasome-mediated degradation of the target protein.

E3 ligases are increasingly being demonstrated to contribute to oncogenesis and have become a promising target in cancer. The development of protein-targeting chimeric molecules (PROTACS) over the last decade has shown potential in targeting proteins including; c-MET, MCL-1, MYC and TRIM24, which were once deemed undruggable [115]. PROTACS recruit and link E3 ligases to a protein of interest by acting as a bridge between the enzyme and substrate. The protein is then modified with K48-linked ubiquitin chains and is, subsequently, degraded by the 26S proteasome. The mode of action of PROTACS is depicted in Figure 5. This manipulation of the UPS has the potential to find, bind and reduce the abundance of oncogenic proteins within the cell, thereby eliciting an anti-cancer effect [116,117,118]. PROTACS targeting MDM2 and PCNA have recently been described, highlighting the potential of this approach in targeting genomic instability [119,120]. While further research into PROTACS is required to ensure clinical efficacy and safety, their ability to effectively degrade hard to target proteins and potential in overcoming drug resistance will no doubt gain them entry to the arsenal of anti-cancer treatments in the future.

## 6. Conclusions and Future Perspectives

Coordination of DNA replication is paramount to maintaining genome stability including origin firing, rescuing stalled forks and termination. The cell has developed many cellular cascades that respond to DNA damage and replication stress. Their response acts to facilitate repair, cell cycle arrest and, when this does not suffice, apoptosis. Deregulation of the DDR and replisome machinery fuels the genomic instability needed to drive cancer cell development and clonal evolution that affords tumour cells immunity from chemotherapies. In the past, the recognition of the deregulation of members in DNA repair and replication led to the discovery of novel therapeutics such as PARP inhibitors, which highlighted genomic stability as an Achilles heel of tumours with defective DNA repair mechanisms. Now, studies into other mediators of replication fork protection, including RPA and RAD51, their roles in cancer and the development of chemo-resistance are starting to be elucidated. This may set the stage for the development of therapies aimed at the regulators of these proteins in an attempt to manipulate DNA repair pathways by disrupting the abundance of machinery that may help in the fight against drug resistance. With further research into the ubiquitin proteasome system and its role as a regulator of genome stability, it is likely that novel therapies, such as specific small-molecule inhibitors and better defined PROTACS, will emerge.

## Figures and Tables

**Table 1:** Inhibitors of the Ubiquitin Proteasome System in Clinical Development.

Target	Inhibitor	Cancer Type	Clinical Stage	Reference/ Trial ID
20S Proteasome (Beta-5 subunit)	Bortezomib	MM, MCL	Approved	[93,94]
	Carfilzomib	MM	Approved	[95]
	Ixazomib	MM	Approved	[96]
	Oprozomib	MM	Phase Ib/II	[97]
	Marizomib	MM	Phase I	[98]
		DIPG	Phase I	NCT04341311
MDM2	Idasanutlin	Glioblastoma	Phase III	NCT03345095
		Breast Cancer	Phase I/II	NCT03566485
		AML/ALL	Phase I/II	NCT04029688
	AMG-232	Sarcoma	Phase Ib	NCT03217266
		Lymphoma	Phase I	NCT04502394
		MM	Phase I	NCT03031730
		AML	Phase Ib	NCT04190550
	HDM201	CRC	Phase I	NCT03714955
	APG-115	AML	Phase Ib	NCT04275518
CRL	MLN4924 (Pevonedistat)	MM	Phase I	NCT03770260
		AML	Phase Ib	NCT01814826
		AML	Phase I/II	NCT03862157
		AML/MDS	Phase I	NCT03772925
		NSCLC	Phase II	NCT03965689
		Lymphoma	Phase I	NCT03323034
		ALL/NHL	Phase I	NCT03349281

MM: Multiple Myeloma; MCL: Mantle Cell Lymphoma; DPIG: Diffuse Intrinsic Pentine Glioma; AML: Acute Myeloid Leukaemia; ALL: Acute Lymphocytic Leukaemia; CRC: Colorectal Cancer; MDS: Myelodysplastic Syndrome; NSCLC: Non-Small Cell Lung Carcinoma; NHL: Non-Hodgkin’s Lymphoma.
